# Successful en bloc resection of large esophageal hemangioma by endoscopic submucosal dissection: A case report

**DOI:** 10.1097/MD.0000000000022821

**Published:** 2020-10-23

**Authors:** Yan Yu, Bingzheng Shen, Chao Zhang, Jiqiao Zhang, Li Cao, Panpan Lu, Mei Liu

**Affiliations:** a Department of Gastroenterology, Tongji Hospital, Tongji Medical College, Huazhong University of Science and Technology; b Department of Pharmacy, Renmin Hospital of Wuhan University; c Institute of Pathology, Tongji Hospital, Tongji Medical College, Huazhong University of Science and Technology, Wuhan; d Department of Gastroenterology, Minda Hospital of Hubei Minzu University, Enshi City, China.

**Keywords:** endoscopic submucosal dissection, esophageal hemangioma

## Abstract

**Rationale:**

With the development of endoscopic techniques, endoscopic therapy began to play an important role in the management of esophageal hemangiomas.

**Patients concerns:**

A large esophageal submucosal tumor (2.5 cm), which was suspected to be an esophageal hemangioma, was diagnosed in a 50-year-old woman.

**Diagnosis:**

Esophageal hemangioma

**Interventions:**

Endoscopic submucosal dissection was performed for tumor removal.

**Outcomes:**

Histopathological results revealed hemangioma. No complication or recurrence was observed in the 17-month follow-up period.

**Lessons:**

Our successful experience showed that endoscopic submucosal dissection is an effective and a safe approach to treat large esophageal hemangiomas (2.5 cm).

## 1 Introduction

Esophageal hemangiomas are uncommon benign vascular tumors.^[1]^ Esophagectomy is the conventional surgical approach for treatment of esophageal hemangiomas; however, recently less invasive approaches are widely used.^[2,3]^ Argon plasma coagulation,^[4]^ endoscopic sclerotherapy,^[5]^ and endoscopic mucosal resection (EMR)^[6]^ are preferred for the treatment of small lesions (<2 cm). Furthermore, endoscopic sclerotherapy alleviates discomfort of larger lesions (>2 cm) with high recurrence rates.^[5]^ One Previous study showed that endoscopic submucosal dissection (ESD)may be applied for esophageal hemangiomas (>2 cm).^[1]^ We herein, report a case of a large submucosal esophageal hemangioma (2.5 cm) in a 50-year old patient that was successfully removed en bloc by ESD.

## 2 Case presentation

**Figure 1:**
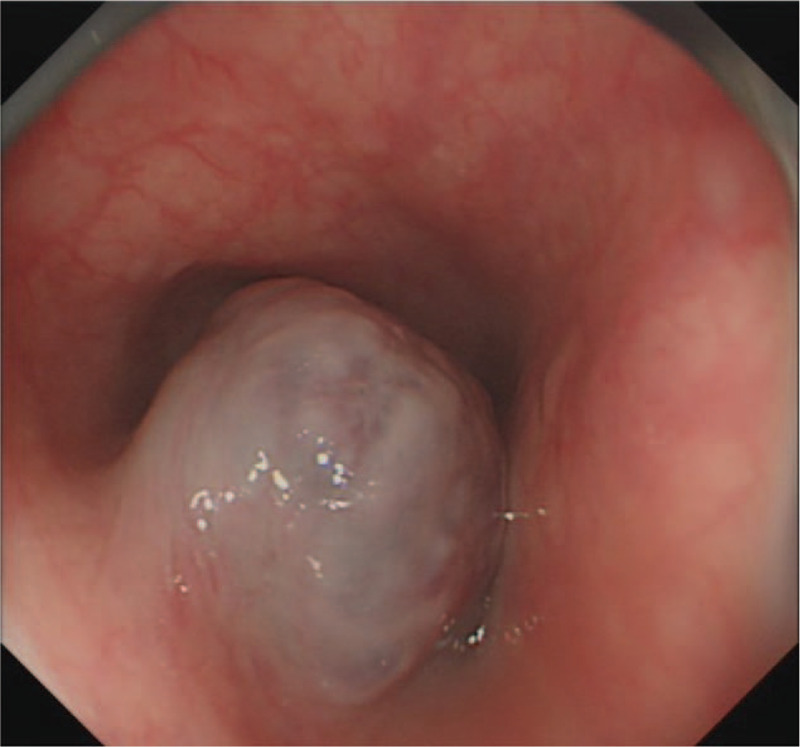
Endoscopy showing edapale-bluish and engorged polypoid mass protruding from esophageal wall in the vertical direction in the upper esophagus.

**Figure 2:**
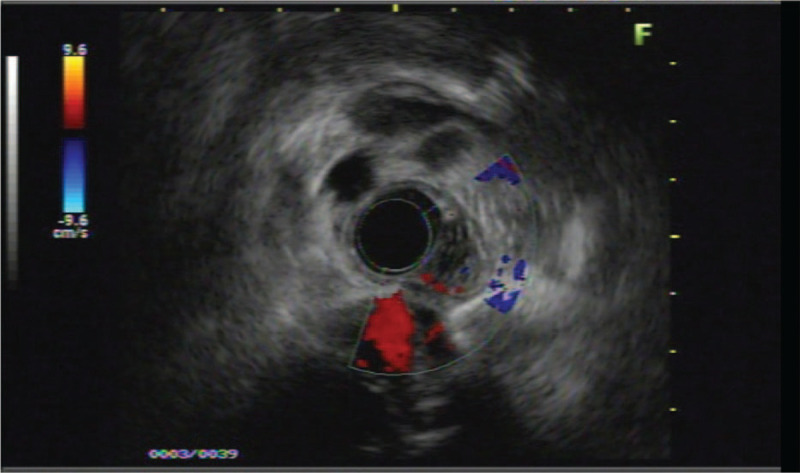
Endoscopic ultrasonography (EUS) using a 12-MHz probe revealed well-defined tumor with heterogenous echogenicity within the submucosa.

**Figure 3:**
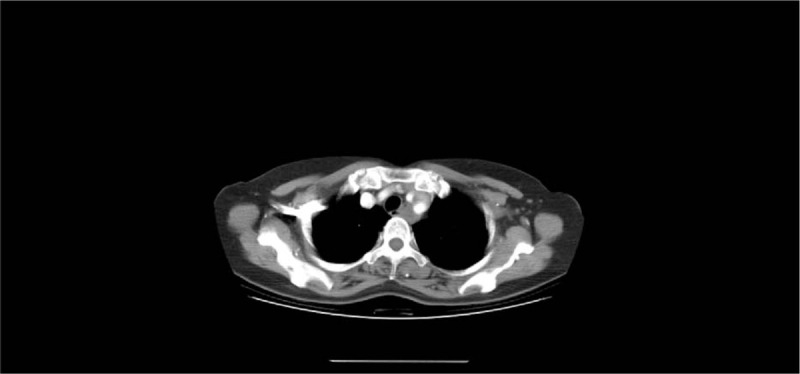
Contrast-enhanced computed tomography (CT) of the thoracic region revealed well-defined luminal protruding nodule at the upper-esophagus.

**Figure 4:**
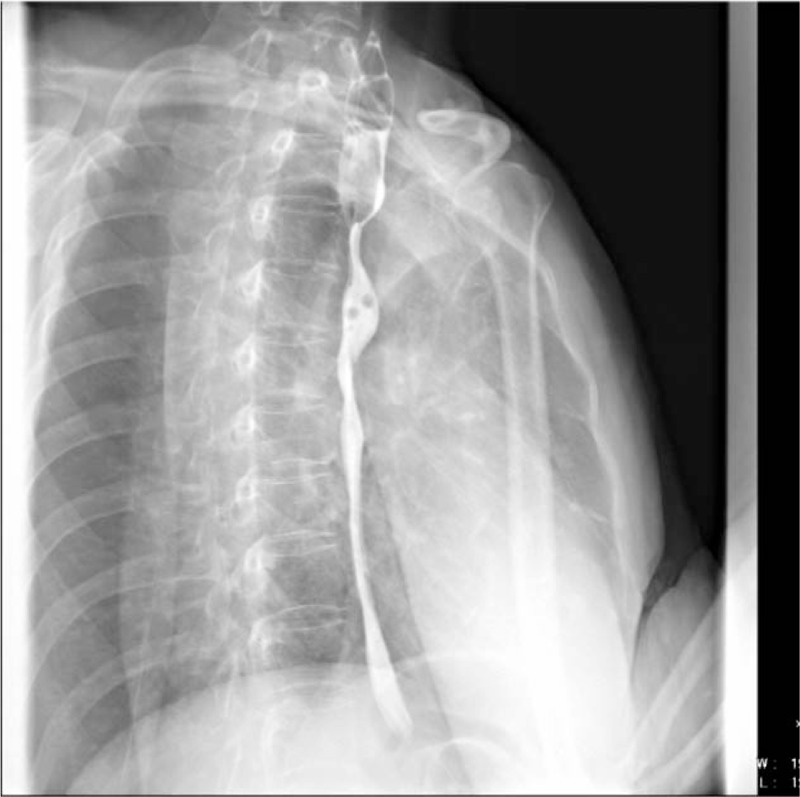
Iodine hydrography revealed a smooth, oval-shaped filling defect and stenosis in upper esophageal lumen.

A 50-year-old woman presented with a 4-month history of dysphagia. The patient's physical and overall nutritive conditions were good. No abnormal physical findings in the thoracic or abdominal region were observed. No abnormalities were detected in the peripheral blood, blood biochemistry, results of blood coagulation tests, tumor markers, and viral markers. The patient underwent upper gastroesophageal endoscopy, which revealed a 10 mm diameter pale-bluish and engorged polypoid mass obstructing the esophagus 18-21 cm from the central incisors (Fig. 1). On endoscopic ultrasonography (EUS), the lesion exhibited as a hypoechoic mass with numerous irregular anechoic spaces localized within the submucosal layer (Fig. 2). Contrast-enhanced computed tomography of the thoracic region revealed a well-defined luminal protruding nodule in the upper esophageal region that did not invade the surrounding organs (Fig. 3). Further, iodine hydrography showed a smooth, oval-shaped filling defect and stenosis in the upper esophageal lumen (Fig. 4). Written informed consent was obtained from the patient for publication of this case report and accompanying images. ESD was performed for diagnostic purpose. This study was approved by the Ethics Committee of Tongji Hospital, Tongji Medical College, Huazhong University of Science and Technology (ethical approval no. TJ-IRB20191212).

**Figure 5:**
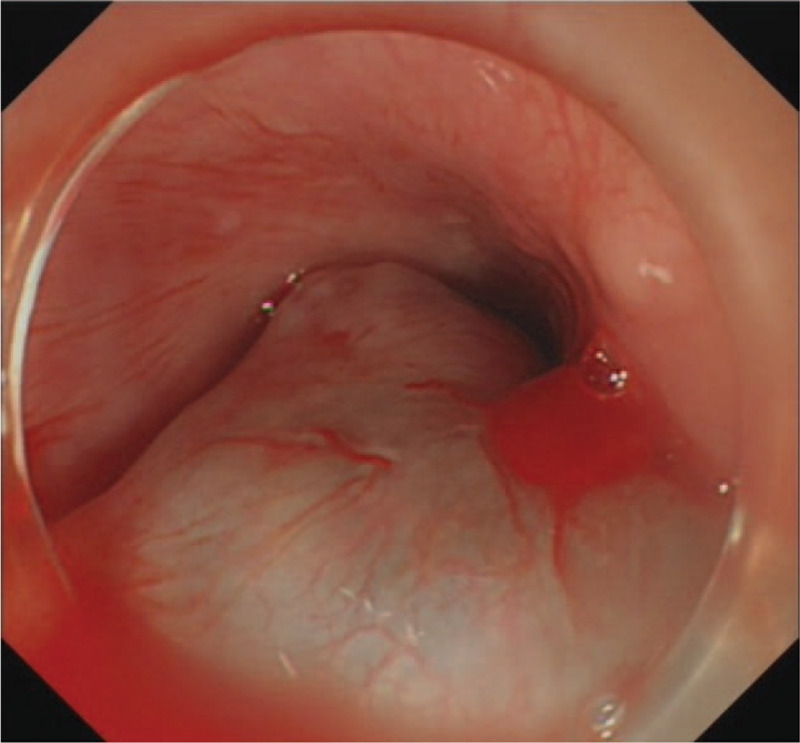
Submucosal injection.

**Figure 6:**
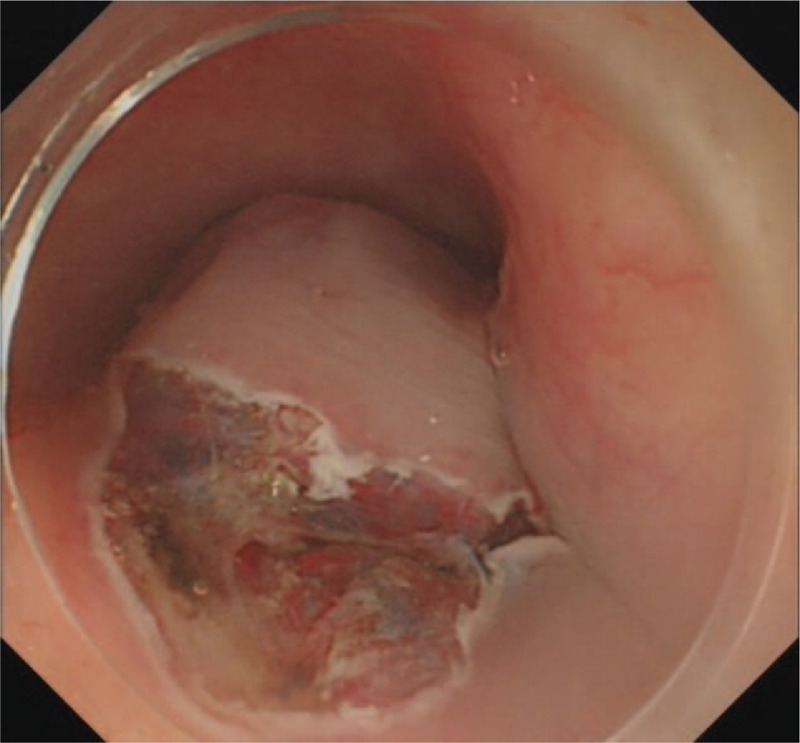
Procedure of the ESD. Circumferential incision. ESD = endoscopic submucosal dissection.

**Figure 7:**
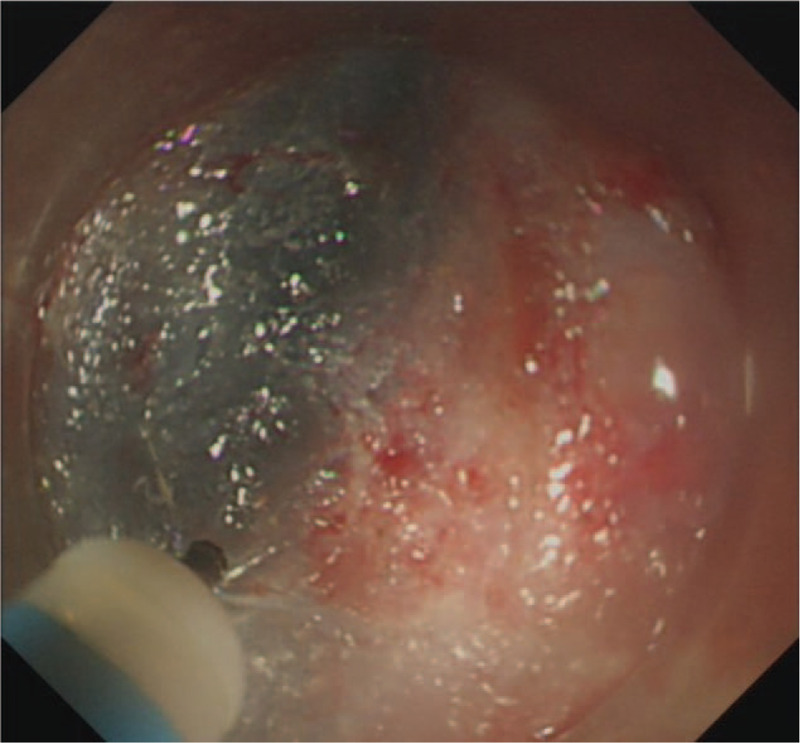
Procedure of the ESD. Active incision. ESD = endoscopic submucosal dissection.

**Figure 8:**
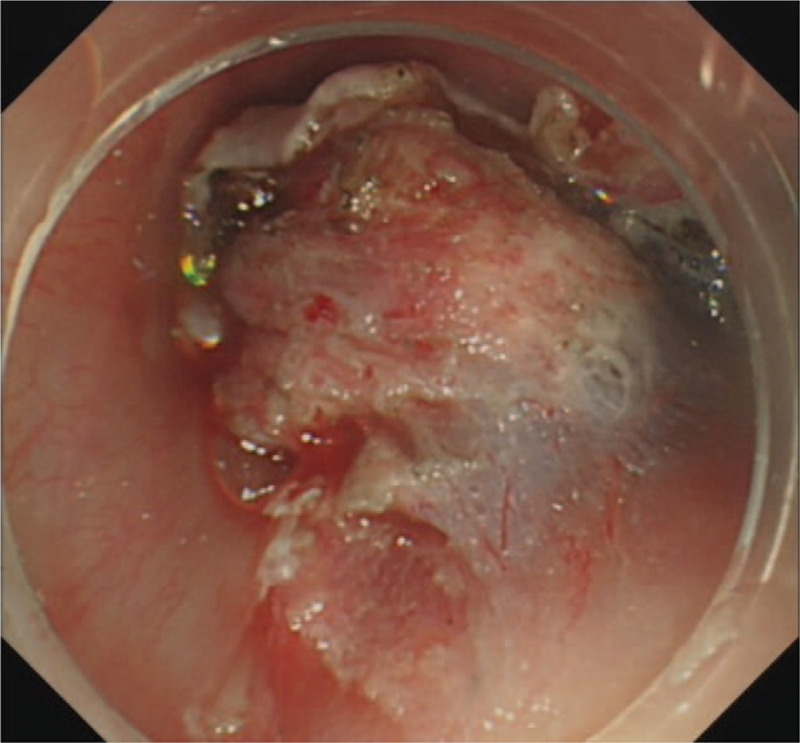
Procedure of the ESD. Unroofing to expose the tumor. ESD = endoscopic submucosal dissection.

**Figure 9:**
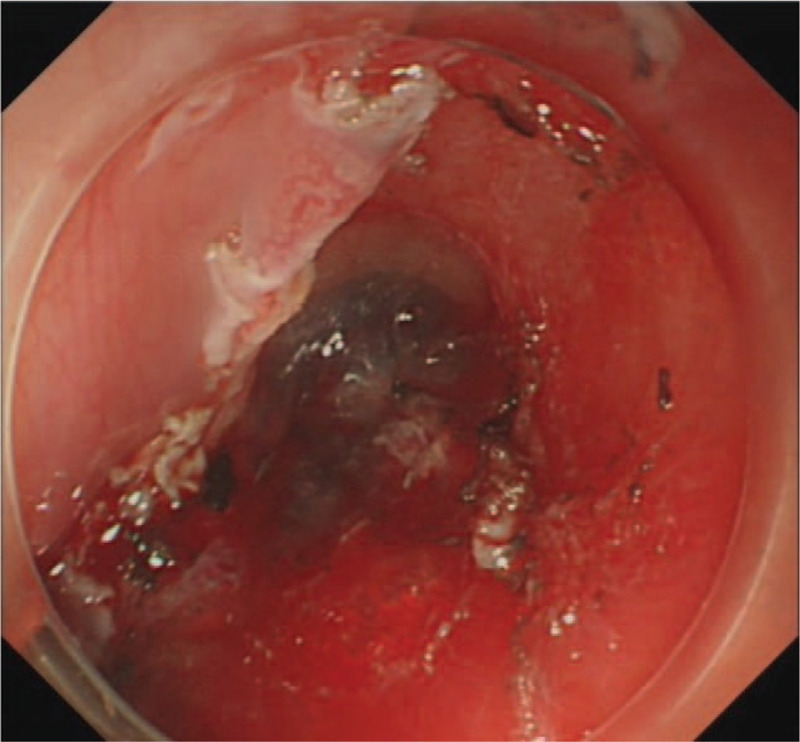
Procedure of the ESD. The wound after ESD. ESD = endoscopic submucosal dissection.

ESD was performed under general anesthesia. The first submocosal injection was administered 0.5 cm away from the lesion to avoid bleeding. In the process of incision, the transparent cap was fixed on the head of the gastroscope (GIF HQ 190, Olympus). The front end of transparent cap was approximately 0.3 cm away from the front end of the gastroscope, which helped to partially peel the lesion and widened the operative field. This cap prevented the tip of the dual knife (KD-650Q, Olympus) to be too close to the mass. The distance between the first incision and hemangioma was approximately 0.5 cm. Following the incision of the mucosal layer, the submucosal layer was dissected from the oral side using a dual knife (Fig. 5). The correct submucosal plane directly above the muscular layer was identified, which enabled dissection of the target layer (Figs. 6–8). The outer membrane of the hemangioma was kept away from the dissection site. The large blood vessels were avoided or pre-coagulated by the coagulation forceps (Coagrasper, FD-411QR, Olympus), before dissection to prevent intraoperative bleeding (Fig. 9). As a clear operative field was maintained for ESD, the dissection was performed without disturbing the hypervascular hemangioma. Besides, the transparent cap could lift the lesion and expand the surgical field, enabling the knife to be more flexible and keeping it away from the muscular layer of the esophagus. Hence, the perforation was avoided.

**Figure 10:**
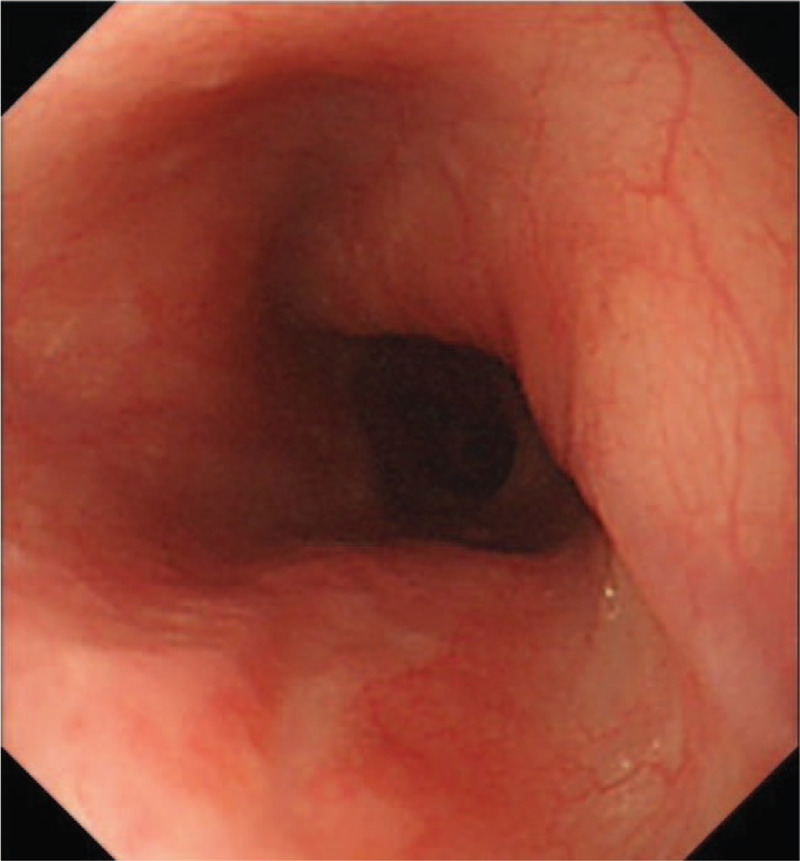
After 3 mo, a follow-up endoscopy showed no recurrence or complication at the ESD site. ESD = endoscopic submucosal dissection.

**Figure 11:**
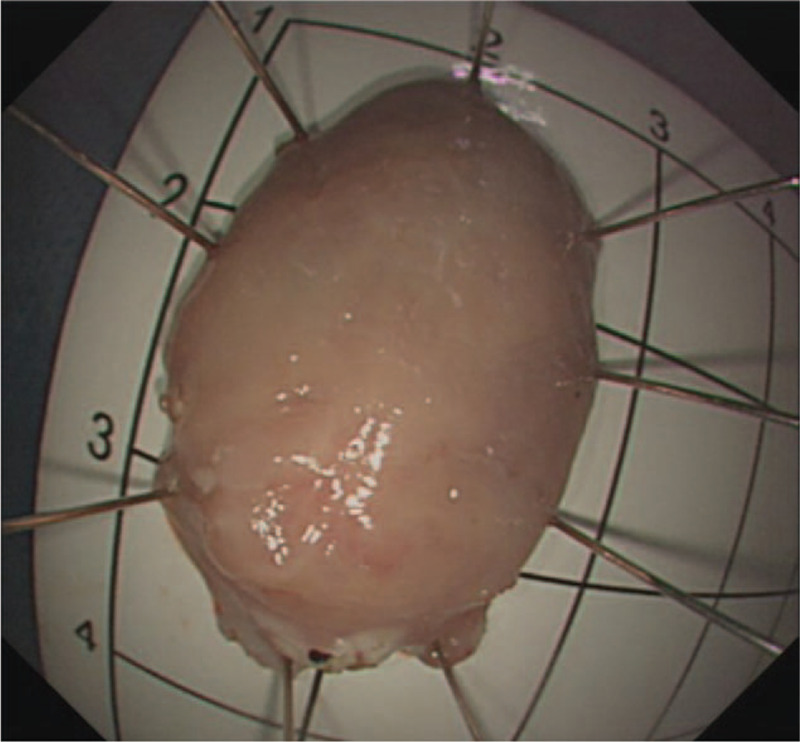
Macroscopic appearance of the resected specimen.

**Figure 12:**
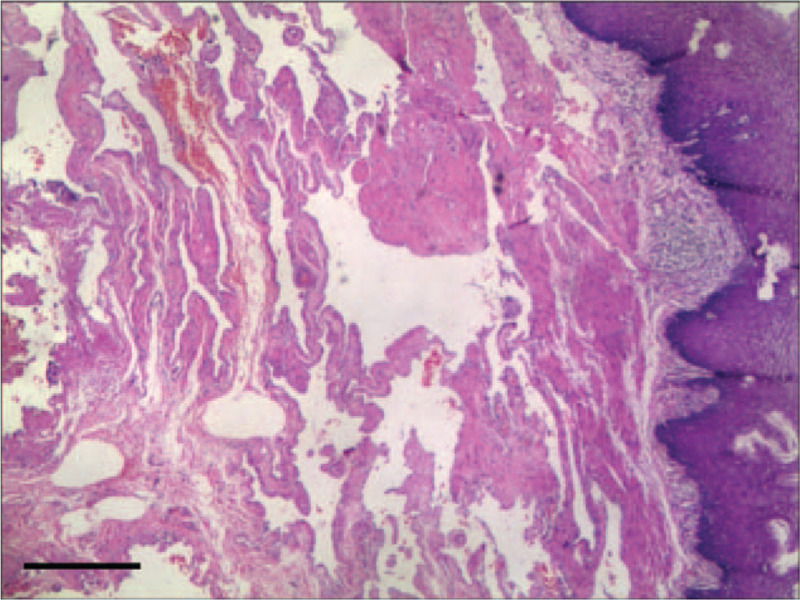
Histopathologic findings showed a vascular lumen with irregular dilatation below the muscularis mucosa, suggesting esophageal cavernous hemangioma (orig. mag. 400, bar = 50 μm).

**Figure 13:**
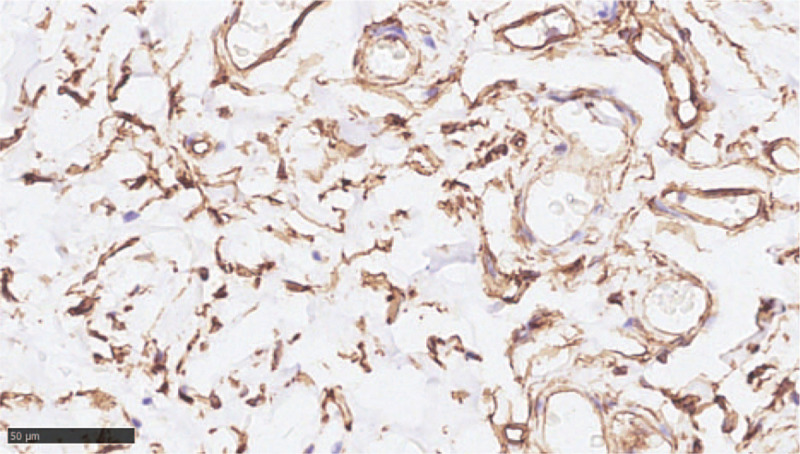
Immunohistochemistry staining of CD31 of the mass (orig. mag. 400, bar = 50 μm).

No complication occurred after ESD. A broad-spectrum antibiotic and proton pump inhibitor were administered intravenously 1 hour after the surgery and for the next 3 days. The patient was fasting and receiving fluid therapy for 3 days. She was discharged 5 days after the surgery and an oral proton pump inhibitor was prescribed for the next 3 weeks. After 3 months, a follow-up endoscopy was performed that showed no recurrence or complication at the ESD site (Fig. 10). The patient did not exhibit any recurrence for 17 months. The histopathological findings of the resected specimen measuring 2.5 cm × 1.5 cm × 0.5 cm showed a vascular lumen with irregular dilatation below the muscularis mucosa, indicating an esophageal cavernous hemangioma (Figs. 11 and 12). Immunohistochemical staining of the cluster of differentiation 31 was positive, which was consistent with the diagnosis (Fig. 13).

## 3 Discussion

Esophageal hemangiomas are uncommon benign vascular tumors,^[1]^ which represent approximately 3% of all benign esophageal tumors.^[7]^ Typically, the overlying bluish mucosa can be easily detected in the esophagus. On computed tomography, esophageal hemangiomas appear as homogenous and isodense mass with calcifiation.^[8]^ On EUS, the hemangioma appears hypoechoic and involves the mucosal and submucosal layers with intact muscularis propria.^[9]^ In our case, EUS was performed to detect the depth of the lesion and to evaluate the risk of perforation during ESD. In most cases, asymptomatic hemangiomas require only clinical observation. However, patients with symptoms, such as bleeding from the hemangioma and digestive tract stenosis, may require treatment.^[9]^ Esophagectomy is the conventional surgical approach for treatment of esophageal hemangiomas; however, recently less invasive approaches, such as endoscopic therapy, are widely used.^[2,3]^

A previous study reported a case of a small esophageal hemangioma (<0.5 cm) that was successfully treated by repeated argon plasma coagulation.^[4]^ Sclerotherapy can also be utilized for the small lesion (<2 cm) with lower risk of recurrence. The common agents include 5% ethanolamine oleate,^[10]^ 1% polydocanol followed by 99% ethanol,^[8]^ and ethanoloamine.^[5]^ Potassium titanyl/yttrium aluminum garnet laser therapy^[11]^ and endoscopic sclerotherapy^[5]^ are less invasive strategies than surgery for large esophageal hemangiomas (>10 cm), which cannot be managed by endoscopic resection and often need repeated treatment to reduce the recurrence of tumor. The mass decreases in size and the obstructive symptoms are alleviated after sclerotherapy.^[5]^ Although endoscopic sclerotherapy may cause hemorrhage and necrosis on the puncture site, no perforation has been reported.^[5,8,10]^ Since sclerotherapy cannot procure specimens for pathological examination and is associated with a risk of residual or recurrent hemangioma,^[5]^ en bloc removal is a better treatment option.

Several techniques have been used to treat esophageal hemangiomas in different sizes for endoscopic en bloc removal. EMR has been widely used in the en bloc resection of small tumors (<2 cm).^[6]^ EMR is less invasive than ESD and may reduce the risk of perforation, and thus, has been used in pedunculated esophageal hemangiomas (<2 cm).^[9,12]^ However, EMR can cause bleeding. Combined EMR with endoscopic band ligation^[13]^ or hemostatic clipping^[2]^ can be performed as alternative treatment options for esophageal hemangiomas with the advantage of less bleeding and perforation risks. However, sometimes repetitive EMR is necessary to prevent recurrence.^[9]^

The number of patients undergoing ESD is increasing, especially in cases of large size tumors (>2 cm).^[14]^ ESD may be used in cases of small and large esophageal hemangiomas (1–2.5 cm) without obvious complications.^[1,15]^ ESD in cases of vascular lesions in the esophagus is risky because of the hypervascularity of the tumor. The risk of severe complications (bleeding and perforation) is equal to the size of the tumor. Herein, we have presented a case of a large submucosal esophageal hemangioma successfully removed en bloc by ESD. Based on this case report, the key point is the avoidance and pre-management of the blood vessels in the wound to maintain a clear operation field. Moreover, our case highlights the benefits of ESD in shortening the recovery period. The benefits of ESD in treating esophageal hemangioma include minimal invasiveness compared to the surgery, low recurrence rate, integrity of esophagus, and short recovery time. The risk of this endoscopic surgery is local complications; hemorrhage and perforation are prone to occur in the large lesions. The limitation of this study is that we did not investigate the small and large intestine to detect hemangiomas in this patient.

In conclusion, the best endoscopic treatment for esophageal hemangioma should be effective, safe, and have a low recurrence rate and few complications. We suggest that appropriate submucosal injection, electrocoagulation hemostasis, and maintaining a clear operative field could contribute to successful ESD procedures. Thus, ESD may be utilized for esophageal hemangiomas. Further, the benefits and risks of ESD in the treatment of esophageal hemangiomas (>2.5 cm) need to be investigated in the future.

## Author contributions

**Conceptualization:** Yan Yu, Mei Liu.

**Data curation:** Yan Yu, Mei Liu.

**Formal analysis:** Yan Yu, Bingzheng Shen, Mei Liu.

**Funding acquisition:** Yan Yu.

**Investigation:** Yan Yu.

**Methodology:** Jiqiao Zhang, Mei Liu.

**Project administration:** Yan Yu, Mei Liu.

**Resources:** Chao Zhang, Mei Liu.

**Software:** Bingzheng Shen, Li Cao.

**Supervision:** Mei Liu.

**Validation:** Bingzheng Shen.

**Visualization:** Chao Zhang, Panpan Lu.

**Writing – original draft:** Yan Yu.

**Writing – review & editing:** Yan Yu, Bingzheng Shen, Mei Liu.
